# Long-Term Follow-Up of Lower Urinary Tract Outcome in Children with Dysfunctional Voiding

**DOI:** 10.3390/jcm11247395

**Published:** 2022-12-13

**Authors:** Chung-Hsin Peng, Sheng-Fu Chen, Hann-Chorng Kuo

**Affiliations:** 1 Department of Urology, Cardinal Tien Hospital, School of Medicine, Fu-Jen Catholic University, New Taipei City 242062, Taiwan; 2 Department of Urology, Hualien Tzu Chi Hospital, Buddhist Tzu Chi Medical Foundation, Tzu Chi University, Hualien 970473, Taiwan

**Keywords:** voiding dysfunction, urinary incontinence, urinary tract infection, vesicoureteral reflux

## Abstract

**Objective:** To investigate the long-term clinical and urodynamic outcomes of a small cohort of children who received short-term urotherapy for confirmed dysfunctional voiding (DV) and lower urinary tract symptoms (LUTS). **Materials and Methods:** This study included 26 children with confirmed LUTS and DV via video urodynamic study (VUDS) and received standard urological therapy, pelvic floor muscle training, or surgical intervention in childhood. Their current lower urinary tract conditions were assessed by chart review and direct and telephone interviews. Charts of 14 patients who underwent follow-up VUDS were reviewed to investigate their bladder and voiding dysfunction or follow-up on previous treatment results. The satisfaction of lower urinary tract status was assessed using the global response assessment (GRA) scale. **Results:** At initial enrolment, the mean age was 9.54 ± 3.88 years, and urological treatment was performed during the first 1–5 years thereafter. Most patients were not regularly followed in the urology clinic. Among the 14 children available for follow-up, a GRA score of 3 was reported by 10 (71.4%) after a mean follow-up period of 10.3 ± 6.74 (range, 2–20) years, indicating satisfactory bladder and voiding conditions. Four children with less favorable outcomes (GRA score of <3) had significantly more post-void residual urine volume at baseline, and 75% of these patients had central nervous system diseases. Daytime incontinence and enuresis rates were significantly decreased at follow-up compared with the baseline. Significantly increased bladder capacity and sensation and significantly decreased voiding detrusor pressure were found on follow-up VUDS. **Conclusions:** Children with DV who received standard urotherapy upon diagnosis exhibited improved LUTS at a 10-year follow-up. Of the 14 children available for follow-up, 10 (71.4%) reported satisfactory bladder and voiding status without further medication or urotherapy, with significantly decreased voiding detrusor pressure.

## 1. Introduction

Dysfunctional voiding (DV) in children may result in lower urinary tract symptoms (LUTS), such as urgency, frequency, urinary incontinence, and difficulty in urination. High post-void residual (PVR) urine volume, vesicoureteral reflux (VUR), and recurrent urinary tract infection (UTI) may develop in children with severe DV, which can lead to renal function deterioration in the absence of appropriate treatment [1]. Detrusor overactivity (DO) is the most common urodynamic finding in children with DV, including high voiding detrusor pressure, recurrent UTI, bilateral VUR, and bowel dysfunction [2]. We previously reported DV in 75.7% of children with LUTS and urodynamic DO, 73.3% with VUR, 63% with urinary incontinence, 77% with UTI, and all children with diurnal enuresis [3].

DV is associated with increased disease burden, and it reduces the quality of life in children with urinary incontinence, recurrent UTI, and VUR; therefore, comprehensive patient management is recommended for the early diagnosis and treatment of pediatric DV. Noninvasive assessments and biofeedback-based pelvic floor muscle training (PFMT) can effectively improve LUTS and protect upper urinary tract function in children, in addition to medical treatment [4,5]. The standard urotherapy includes regular optimal voiding regimens and bowel programs, in combination with prudent use of antimuscarinics and α-blockers have been used for treatment of children with overactive bladder and DV [6]. Adding biofeedback-based PFMT to standard urotherapy significantly improved the therapeutic outcomes [7].

Long-term outcomes were seldomly reported although studies have investigated the diagnosis and treatment outcomes of DV in children. The pathophysiology of non-neurogenic DV is associated with a delayed functional lower urinary tract maturation, resulting in inadequate urethral sphincter relaxation during involuntary detrusor contractions. Lower urinary tract dysfunction in children may improve and the LUTS may disappear over time in parallel with developmental growth [6]. The present study investigated the long-term clinical and urodynamic outcomes in a small cohort of children who received short-term therapy for confirmed DV and LUTS in childhood to identify factors for good clinical outcomes.

## 2. Materials and Methods

This cohort study included 26 females with LUTS who received urological therapy in childhood. The clinical presentation and the video urodynamic study (VUDS) results of the cohort were previously reported [3]. The present study performed chart review and direct and telephone interviews to assess the current lower urinary tract condition of the 26 patients after a mean follow-up period of 10.3 ± 6.74 (range, 2–20) years. Additionally, based on the chart review, 14 patients underwent follow-up VUDS to investigate bladder and voiding function and assess previous treatment outcomes. The study was approved by the Research Ethics Committee of Hualien Tzu Chi Hospital, Buddhist Tzu Chi Medical Foundation (approval no.: 110-147-B). Informed consent was waived due to the retrospective study design.

VUDS was performed with patients in the awake state. The pressure-flow study was performed using a 4.5-Fr dual-lumen catheter, and C-arm fluoroscopy was concomitantly used to visualize and assess the lower urinary tract condition during the storage and voiding phases [3]. VUDS was routinely performed at study enrolment and was repeated postoperatively, with noninvasive urotherapy, or bladder management. The guidelines of good urodynamic practices by the International Continence Society recommended VUDS as a standard procedure [8]. Patients who could not move to the examination chair were assessed in the supine position, and the pressure-flow study was performed under fluoroscopic examination. Data on the first bladder filling sensation, full bladder sensation, urge sensation, cystometric bladder capacity, bladder compliance, presence of DO, maximum flow rate (Qmax) during voiding, detrusor pressure at Qmax (Pdet) during voiding, voided volume, and PVR volume were recorded. The bladder neck opening and narrowing, urethral sphincter dyssynergia, poor external sphincter relaxation, and distal urethral narrowing were assessed by performing bladder and bladder outlet imaging during voiding. Abdominal leak point pressure was determined upon urine leakage during abdominal pressure at bladder capacity without detrusor contractility. The bladder contractility index was calculated as follows: bladder contractility index = Pdet + (5 × Qmax); the voiding efficiency as voiding efficiency = voided volume/voided volume + PVR; and the bladder outlet obstruction index as bladder outlet obstruction index = Pdet.Qmax − (2 × Qmax). Additionally, bladder contour, the presence and grade of VUR, and cystourethrographic findings during the bladder storage and emptying phases were assessed.

**Figure 1:**
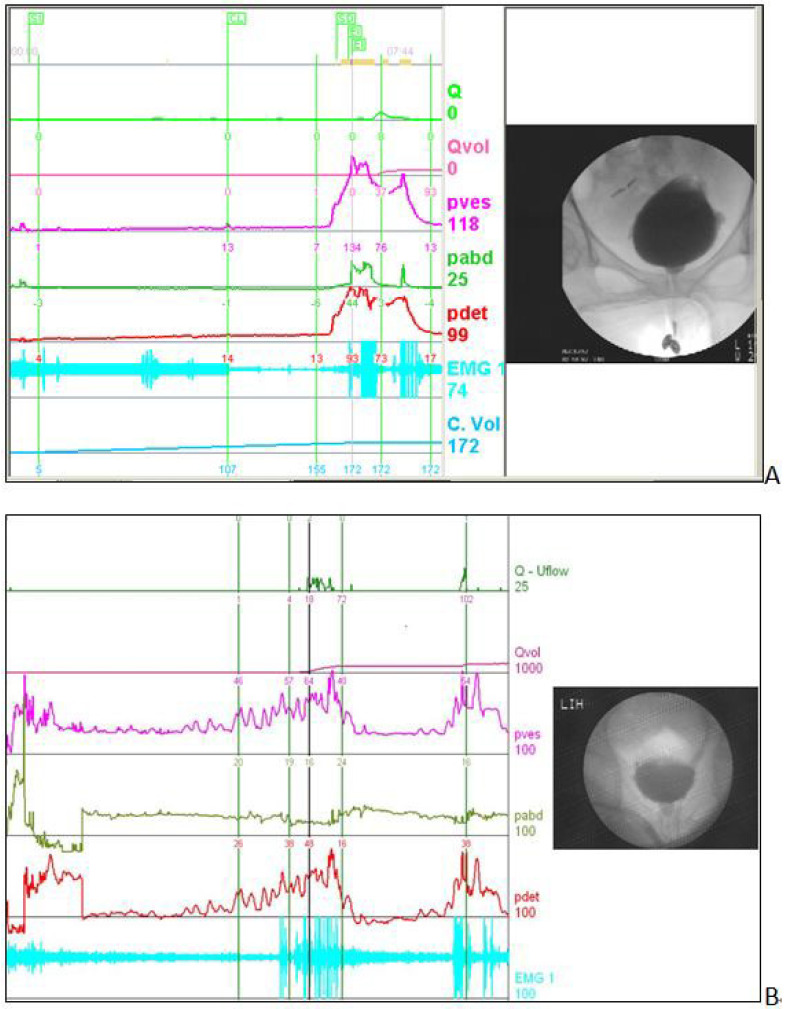
Video urodynamic tracings of children with dysfunctional voiding. (**A**) shows the tracing of a patient with high voiding pressure, low flow rate, increased urethral sphincter electromyographic activity, and tight urethra sphincter during voiding. (**B**) shows the tracing of a patient with detrusor overactivity during bladder filling, with high detrusor pressure, tight urethral sphincter, and right vesicoureteral reflux during voiding. Q, flow rate; Qvol, voided volume; Pve, intravesical pressure; Pabd, intra-abdominal pressure; Pdet, detrusor pressure; EMG, electromyogram of external sphincter.

DV diagnosis was based on the presence of sustained high Pdet, low Qmax, non-relaxing or increased electromyographic activity of the urethral sphincter, with or without a large PVR volume, and dilated proximal urethra and narrow mid-urethra during voiding (Figure 1) [3]. Children with spinal bifida occulta, myelomeningocele, and spinal cord lesion induced neurogenic bladder were not included. The control group included 10 age-matched patients with diurnal urinary incontinence or voiding dysfunction and referred for urological investigation. These children were found to have a normal lower urinary tract function without evidence of DV based on VUDS. The data of the control children were used for comparison of the VUDS parameters in children with DV.

All patients were treated with antimuscarinics, α-blockers, or baclofen for DO and urethral sphincter hyperactivity after confirming DV. Additionally, children older than 5 years, who were able to follow the instructions and perform PFMT, underwent biofeedback-based PFMT. Unilateral or bilateral ureteral reimplantation had been performed in patients who developed frequent UTI, high-grade VUR, and hydronephrosis before initiation of urotherapy for DV. Repeated urethral injections of botulinum toxin A (Botox^®^) were performed at 6-month intervals in those with severe DV and large PVR urine volume. Intravesical Botox^®^ was also performed in patients with refractory urinary incontinence.

Patients were followed up in the urological clinic for several years after treatment initiation, regardless of continuous medical or noninvasive treatment. Patients with episodic UTI received antibiotic treatment, and those with voiding dysfunction received standard urotherapy. Biofeedback-based PFMT was discontinued in most patients after active management in the first few years. Chart review and direct or telephone interview were performed to assess the LUTS and bladder conditions, and the global response assessment (GRA) scale was used by the patients to grade their current bladder and voiding conditions as follows: 0, no improvement; 1, mild improvement; 2, moderate improvement; and 3, marked improvement. A GRA score of 3 was considered a good clinical outcome and a score of <3 was considered a less favorable outcome. The baseline and follow-up LUTS, bladder conditions, and VUDS parameters were compared.

Wilcoxon’s rank-sum test was used to compare continuous variables expressed as means with standard deviation. The chi-square test was used to compare categorical variables expressed as numbers and percentages. All statistical assessments were two-sided, and a *p*-value of <0.05 was considered statistically significant. All statistical analyses were performed using the Statistical Package for the Social Sciences software for Windows (version 16.0; Chicago, IL, USA).

## 3. Results

The mean age at enrolment was 9.54 ± 3.88 years in the entire study cohort of 26 patients with DV. The mean age was 9.70 ± 3.25 years in the controls.The reported LUTS included frequency, urgency, daytime incontinence, enuresis, voiding difficulty, and urine retention in 14 (53.8%), 15 (57.7%), 15 (57.7%), 10 (38.5%), 6 (23.1%), and 1 (3.8%) patients, respectively. Among the children with DV, 4 had central nervous system (CNS) disorders including mild cerebral palsy (*n* = 2), hypoxic encephalopathy (*n* = 1), and epilepsy (*n*= 1); the other 22 children were neurologically normal at enrolment. Additionally, constipation or encopresis, recurrent UTI, VUR, and hydronephrosis were reported in 20 (76.9%), 6 (23.1%), 8 (30.8%), and 6 (23.1%) patients, respectively. Table 1 shows the baseline demographics of the the urodynamic assessment results of 26 patients with DV. Among the patients urgency and urgency urinary incontinence was noted in 18 (69.2%), and difficult urination was noted in 6 (23.1%). Urodynamic DO was highly prevalent in patients with DV (88.5%) (Table 2). Further, the Pdet was significantly higher in patients with DV than in the controls (50.9 ± 23.9 vs. 17.5 ± 6.98 cmH_2_O, *p* < 0.0001). The voiding efficiency was lower and the bladder outlet obstruction index was higher in patients with DV than in the controls, indicating higher bladder outlet resistance in patients with DV.

All patients were managed with invasive or noninvasive urotherapy, including antimuscarinics or beta-3 adrenoceptor agonists for DO, α-blockers with skeletal muscle relaxants for dysuria, and antibiotic treatment and prophylaxis for recurrent UTI. Additionally, urethral Botox^®^ injection, detrusor Botox^®^ injection, and biofeedback-based PFMT were performed in 3, 2, and 11 patients, respectively. All treatments were performed during the first 1–5 years after the diagnosis.

Most patients were not regularly followed up in the urology clinic. The present study followed up with patients using chart review, outpatient visits, or telephone interviews. In the entire cohort of 26 patients, 3 died because of nonurological disease, 2 received bladder augmentation or urinary diversion, and 7 were lost to follow-up. Therefore, the current study evaluation included the remaining 14 patients. Table 3 presents the baseline and follow-up LUTS and urinary tract conditions. Briefly, the rates of daytime incontinence and enuresis were significantly decreased compared with the baseline at the time of initial DV diagnosis. However, some patients still experienced frequency, urgency, voiding difficulty, constipation, and recurrent UTIs.

The overall treatment outcome evaluation revealed that 10 (71.4%) patients had a GRA score of 3, indicating satisfactory bladder and voiding conditions. Patients with a less favorable outcome, i.e., those with a GRA score of <3, have significantly higher PVR urine volume at baseline, and 75% of these patients have associated central nervous system (CNS) diseases, including intracranial tumors (*n* = 2) and hypoxic encephalopathy (*n* = 1) (Table 4). All three patients with CNS diseases and with a GRA score of <3 had persistent constipation or encopresis during the long-term follow-up.

**Figure 2:**
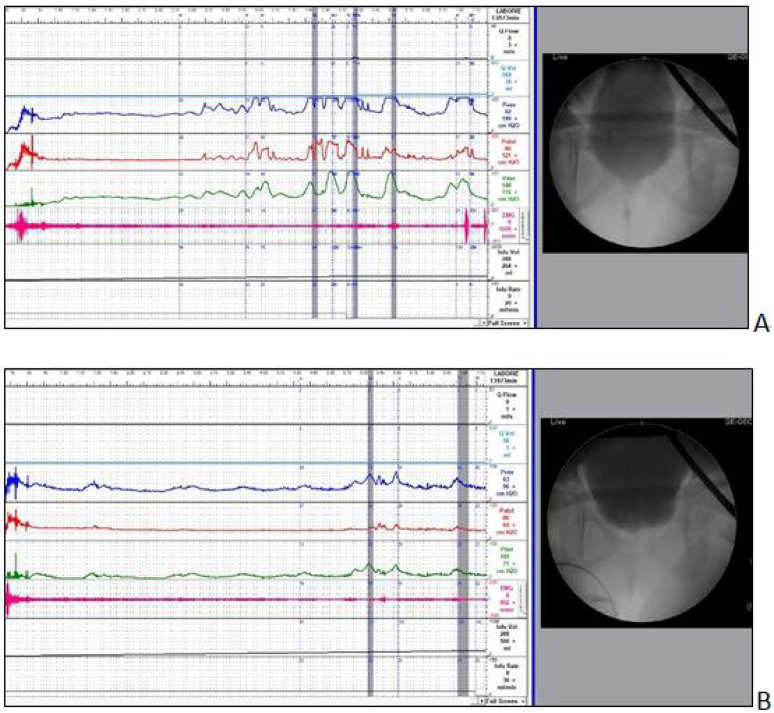
Video urodynamic tracings of a female with dysfunctional voiding and recurrent urinary tract infection. (**A**) Video urodynamic study at baseline shows detrusor overactivity with high voiding pressure and tight urethral sphincter during voiding. (**B**) One year after antimuscarinic treatment, the detrusor overactivity is decreased in frequency and amplitude and the bladder capacity increased, resulting in urinary incontinence improvement. Q, flow rate; Qvol, voided volume; Pve, intravesical pressure; Pabd, intra-abdominal pressure; Pdet, detrusor pressure; EMG, electromyogram of external sphincter.

Follow-up VUDS was performed in the 14 patients at a median period of 3 years after the initial diagnosis (range, 2–20 years) for regular follow-up or persistent LUTS (Figure 2). Table 5 shows the changes in urodynamic parameters at baseline and follow-up. Briefly, the bladder capacity and bladder sensation were significantly increased and the Pdet was significantly decreased (55.8 ± 21.9 vs. 36.1 ± 18.2 cmH_2_O, *p* = 0.039) at follow-up VUDS. However, DO remained present in 42.9% of patients, and the Pdet at follow-up remained higher than that in the controls (36.1 ± 18.2 vs. 17.5 ± 6.98 cmH_2_O, *p* = 0.01). All 14 patients had been off medication and follow-up at the outpatient clinic. They had learned management strategies, such as taking antibiotics or performing clean intermittent self-catheterization to empty their bladder, although 14.3% of the patients still experienced episodic UTI or difficulty in urination.

## 4. Discussion

The present study investigated the long-term treatment outcomes of children with DV who received initial standard urotherapy and noninvasive treatment for LUTS and urological complications. Of the 14 patients who were available for follow-up 10 years after the initial diagnosis, 10 (71.4%) reported satisfactory bladder and voiding conditions, which did not require further medication or urotherapy, and exhibited significantly decreased voiding Pdet.

Children with urgency, frequency, and incontinence have highly prevalent DV [3]. Frequent detrusor contractions during bladder storage may cause the pelvic floor or the urethral sphincter muscles to become overactive, resulting in a tight bladder outlet during voiding [9]. The urethral sphincter and trigonal smooth muscle interact simultaneously; therefore, children with DV may also develop VUR during voiding, leading to ascending recurrent UTI [10]. A high percentage of children with VUR, DO, or episodic UTI might have associated DV [3]. Therefore, effective DV treatment with medication, biofeedback PFMT therapy, or urethral Botox^®^ injection can subsequently improve urgency, frequency, urinary incontinence, VUR, and recurrent UTI [11,12,13].

DV is considered a learned behavior and developmental abnormality that occurs in response to an adverse event during childhood [14]. Delayed maturation of the CNS-mediated control of voiding inhibition and psychological events have been proposed to explain non-neurogenic voiding dysfunction in children [6,15]. The International Children’s Continence Society defines DV as the habitual contraction of the urethral sphincter or the pelvic floor during voiding and the presence of a staccato pattern with or without an interrupted flow on repeated uroflow in concomitantly recorded electromyography [16].

Undiagnosed and untreated children with DV may have persistent urgency, urinary incontinence, recurrent UTI, and hydronephrosis due to VUR [2,5]. A study reported that untreated DV did not affect the overall spontaneous resolution of VUR [17]. Early DV diagnosis and appropriate standard urotherapy may successfully improve LUTS and urinary tract dysfunction without invasive treatment in most children [4]. Combined pharmacotherapy and standard urotherapy might be necessary for children with more severe DV and those with incontinence episodes [18]. Biofeedback-based PFMT has been recommended as the best noninvasive treatment for children with DV among the various available treatment approaches [1,6,19].

The long-term outcomes of DV were seldomly reported although the treatment outcomes are satisfactory in children with DV. The present study revealed that the LUTS and urodynamic parameters improved without further management after initial urotherapy in most children with DV at a 10-year follow-up. Early DV treatment in childhood by reducing DO, relaxing urethral sphincter hyperactivity, and providing antibiotics to treat UTI may eradicate the continuous bladder afferent input and the guarding responses of the urethral sphincter and pelvic floor muscles, which may have long-term therapeutic effects on lower urinary tract dysfunction [6]. However, some of the study participants with DV still had LUTS, recurrent UTI, constipation, and urodynamic DO, suggesting that DV may not be completely cured in some patients after long-term follow-up. The persistence of these symptoms may lead to DV in adult females.

Moreover, the present study revealed that children with associated CNS diseases and large PVR urine volume at baseline might have persistent voiding dysfunction and less favorable outcomes, possibly due to profound neurological deficits. A recent report revealed an overactive bladder in 38.6% of the patients who recovered from childhood LUTS after a median follow-up period of 20 years [20]. Patients with more severe LUTS and incontinence episodes in childhood might be at higher risk for overactive bladder in adulthood. However, whether more aggressive DV treatment in childhood may decrease the incidence of LUTS in adulthood remains unknown and requires further prospective investigation.

The easiest way to measure dysfunctional voiding in children is a validated dysfunctional voiding questionnaire [21]. However, because most of the children were enrolled more than 10 years ago, we did not use this noninvasive symptom score to assess their symptoms. Instead, we used VUDS to investigate the upper and lower urinary tract dysfunction in children with voiding dysfunction and urinary incontinence refractory to conservative treatment. The role of urodynamic studies in diagnosing children with DV remains debatable. The x-ray exposure during VUDS has potential hazard effect to children. One study reported that the VUDS results were not associated with changes in treatment approaches for DV [22]. However, VUDS provides evidence of detrusor function, the presence of VUR, and the bladder neck and urethral sphincter conditions during voiding [23]. VUDS may aid in understanding the pathophysiology of DV and in planning treatment approaches according to the underlying urinary tract dysfunction in children with LUTD [11].

The study limitations include the retrospective analysis of patient data and the assessment of clinical and VUDS outcomes at different time points. Therefore, the VUDS parameters might not represent the true clinical presentation at long-term follow-up. Furthermore, only 14 of the 26 children were available for long-term follow-up, and some patients with satisfactory or less favorable treatment outcomes might have been missed. The final treatment outcome was assessed at a mean of 10 years after the initial treatment period, not immediately after the initial treatment. Therefore, it is not possible to analyze which treatments are more effective for DV. However, the long-term follow-up data presented in the current study have clinical value in the evaluation and management of children with DV.

## 5. Conclusions

Children with DV who received standard urotherapy in the initial period exhibited LUTS improvement at the 10-year follow-up evaluation. Of the 14 children who were available for follow-up, 10 (71.4%) reported satisfactory bladder and voiding conditions without the need for further medication or urotherapy, with a significantly decreased voiding Pdet based on VUDS performed at a median of 3-year follow-up.

## Figures and Tables

**Table 1:** Baseline demographics of the 26 children with dysfunctional voiding.

Clinical Disease or Symptoms	Patient Number (%)
Central nervous system disorder	4 (15.4%)
Recurrent urinary tract infection	6 (23.1%)
Urgency and urgency incontinence	18 (69.2%)
Vesicoureteral reflux	8 (30.8%)
Hydronephrosis	6 (23.1%)
Nocturnal enuresis	11 (42.3%)
Previous ureteral reimplantation	3 (11.6%)
Voiding difficulty	6 (23.1%)
Constipation or Encopresis	20 (76.9%)

**Table 2:** The urodynamic parameters of 26 children with dysfunctional voiding and 10 controls.

Urodynamic Variable	DV (*n* = 26)	Control (*n* = 10)	*p* Value
Age (years)	9.54 ± 3.88	9.70 ± 3.25	
FSF (mL)	59.2 ± 45.2	86.1 ± 54.5	0.308
FS (mL)	97.7 ± 74.6	131 ± 85.0	0.260
CBC (mL)	141 ± 88.6	230 ± 224	0.252
Compliance (mL/cmH_2_O)	38.8 ± 46.8	55.3 ± 54.9	0.374
DO (%)	23 (88.5%)	5 (50.0%)	0.024
Pdet (cmH_2_O)	50.9 ± 23.9	17.5 ± 6.98	0.000
Qmax (mL/s)	13.0 ± 8.95	16.9 ± 8.91	0.247
Volume (mL)	132 ± 90.9	230 ± 224	0.210
PVR (mL)	9.04 ± 13.9	0.00 ± 0.00	0.003
VE (%)	0.90 ± 0.16	1.00 ± 0.00	0.005
BCI	116 ± 47.1	102 ± 48.4	0.439
BOOI	25.0 ± 32.1	−16.3 ± 15.6	0.000

DV: dysfunctional voiding, FSF: first sensation of filling, FS: fullness sensation, CBC: cystometric bladder capacity, DO: detrusor overactivity, Pdet: detrusor pressure, Qmax: maximum flow rate, PVR: post-void residual, VE: voiding efficiency, BCI: bladder contractility index, BOOI: bladder outlet obstruction index.

**Table 3:** Clinical presentations of 14 patients with dysfunctional voiding at baseline and long-term follow-up.

Clinical Presentations	Baseline	Follow-Up	*p* Value
Age (years)	9.64 ± 4.11	19.9 ± 7.41	-
Frequency, *n* (%)	8 (57.1%)	4 (28.6%)	0.125
Urgency, *n* (%)	9 (64.3%)	6 (42.9%)	0.453
Daytime incontinence, *n* (%)	11 (78.6%)	3 (21.4%)	0.008
Enuresis, *n* (%)	8 (57.1%)	1 (7.1%)	0.016
Voiding difficulty, *n* (%)	2 (14.3%)	3 (21.4%)	1.000
Urinary retention, *n* (%)	0 (0.0%)	1 (7.1%)	-
Constipation/Encopresis, *n* (%)	9 (64.3%)	5 (35.7%)	0.125
rUTI/APN, *n* (%)	4 (28.6%)	2 (14.3%)	0.625
VUR/Hydronephrosis, *n* (%)	5 (35.7%)	1 (7.1%)	0.219

rUTI: recurrent urinary tract infection, APN: acute pyelonephritis, VUR: vesicoureteral reflux.

**Table 4:** Urodynamic parameters at baseline between satisfied and unsatisfied patients.

Urodynamic Parameter	GRA < 3 (*n* = 4)	GRA = 3 (*n* = 10)	*p* Value
DO, *n* (%)	4 (100%)	9 (90.0%)	1.000
FSF (mL)	81.0 ± 62.2	43.7 ± 20.3	0.319
FS (mL)	88.5 ± 65.5	71.4 ± 35.9	0.534
US (mL)	108 ± 68.2	84.1 ± 49.9	0.486
CBC (mL)	148 ± 127	134 ± 67.0	0.790
Compliance (mL/cmH_2_O)	68.3 ± 85.8	14.4 ± 9.86	0.298
Pdet (cmH_2_O)	40.0 ± 12.8	51.4 ± 21.6	0.350
Qmax (mL/s)	9.50 ± 8.35	15.8 ± 10.9	0.324
Volume (mL)	128 ± 132	129 ± 67.8	0.976
PVR (mL)	20.0 ± 16.3	4.50 ± 8.32	0.033
VE (%)	0.73 ± 0.28	0.95 ± 0.12	0.057
BCI	21.0 ± 17.0	19.9 ± 33.2	0.952
BOOI	87.5 ± 47.8	130 ± 54.8	0.200
Associated CNS disease	3 (75.0%)	0 (0.0%)	0.011
Family history of urinary	2 (50.0%)	1 (10.0%)	0.065
Constipation/Encopresis (Baseline)	3 (75.0%)	6 (60.0%)	1.000
Constipation/Encopresis (Follow-up)	3 (75.0%)	2 (20.0%)	0.095

GRA: global response assessment, DO: detrusor overactivity, FSF: first sensation of filling, FS: fullness sensation, CBC: cystometric bladder capacity, Pdet: detrusor pressure, Qmax: maximum flow rate, PVR: post-void residual, VE: voiding efficiency, BCI: bladder contractility index, BOOI: bladder outlet obstruction index, CNS: central nervous disease.

**Table 5:** Urodynamic parameters at baseline and long-term follow-up.

Urodynamic Parameter	Baseline	Follow-Up	*p* Value
DO, *n* (%)	13 (92.9%)	6 (42.9%)	0.125
FSF (mL)	61.4 ± 55.0	122 ± 73.6	0.014
FS (mL)	115 ± 108	189 ± 131	0.025
US (mL)	130 ± 121	218 ± 149	0.016
CBC (mL)	146 ± 112	256 ± 155	0.025
Compliance (mL/cmH_2_O)	33.4 ± 31.0	34.3 ± 28.5	0.925
Pdet (cmH_2_O)	55.8 ± 21.9	36.1 ± 18.2	0.039
Qmax (mL/s)	10.3 ± 6.61	10.0 ± 9.33	0.902
Volume (mL)	137 ± 115	167 ± 178	0.458
PVR (mL)	9.44 ± 11.3	88.9 ± 147	0.120
VE (%)	0.90 ± 0.14	0.71 ± 0.44	0.135
BCI	107 ± 44.4	86.1 ± 48.9	0.187
BOOI	35.2 ± 22.3	16.1 ± 27.0	0.054

DV: dysfunctional voiding, FSF: first sensation of filling, FS: fullness sensation, CBC: cystometric bladder capacity, DO: detrusor overactivity, Pdet: detrusor pressure, Qmax: maximum flow rate, PVR: post-void residual, VE: voiding efficiency, BCI: bladder contractility index, BOOI: bladder outlet obstruction index.

## Data Availability

The data are available with the permission of Institutional Review Board after contacting with the corresponding author.
